# Filtration-processed biomass nanofiber electrodes for flexible bioelectronics

**DOI:** 10.1186/s12951-022-01684-3

**Published:** 2022-11-19

**Authors:** Daiki Ando, Tetsuhiko F. Teshima, Francisco Zurita, Hu Peng, Kota Ogura, Kenji Kondo, Lennart Weiß, Ayumi Hirano-Iwata, Markus Becherer, Joe Alexander, Bernhard Wolfrum

**Affiliations:** 1 Neuroelectronics, Munich Institute of Biomedical Engineering, Department of Electrical Engineering, TUM School of Computation, Information and Technology, Technical University of Munich, Hans-Piloty-Str. 1, 85748 Garching, Germany; 2 Medical & Health Informatics Laboratories, NTT Research Incorporated, 940 Stewart Drive, Sunnyvale, CA 94085 USA; 3 Graduate School of Engineering, Tohoku University, 6-6 Aoba, Aramaki, Aoba-Ku, Sendai, Miyagi 980-8579 Japan; 4 New Development Department, Corporate Planning Division, Sugino Machine Limited, 2880 Kuriyama, Namerikawa, Toyama 936-8577 Japan; 5 Nano and Quantum Sensors, Department of Electrical Engineering, TUM School of Computation, Information and Technology, Technical University of Munich, 85748 Garching, Germany

**Keywords:** Bioelectronics, Biopolymers, High-aspect-ratio materials, Membrane filtration, Implantable Electrodes

## Abstract

An increasing demand for bioelectronics that interface with living systems has driven the development of materials to resolve mismatches between electronic devices and biological tissues. So far, a variety of different polymers have been used as substrates for bioelectronics. Especially, biopolymers have been investigated as next-generation materials for bioelectronics because they possess interesting characteristics such as high biocompatibility, biodegradability, and sustainability. However, their range of applications has been restricted due to the limited compatibility of classical fabrication methods with such biopolymers. Here, we introduce a fabrication process for thin and large-area films of chitosan nanofibers (CSNFs) integrated with conductive materials. To this end, we pattern carbon nanotubes (CNTs), silver nanowires, and poly (3,4-ethylenedioxythiophene):poly (styrenesulfonate) (PEDOT:PSS) by a facile filtration process that uses polyimide masks fabricated via laser ablation. This method yields feedlines of conductive material on nanofiber paper and demonstrates compatibility with conjugated and high-aspect-ratio materials. Furthermore, we fabricate a CNT neural interface electrode by taking advantage of this fabrication process and demonstrate peripheral nerve stimulation to the rapid extensor nerve of a live locust. The presented method might pave the way for future bioelectronic devices based on biopolymer nanofibers.

**Supplementary Information:**

The online version contains supplementary material available at 10.1186/s12951-022-01684-3.

## Introduction

Bioelectronics for physiological recordings, biochemical assessments, and electrical modulation is an emerging technology in the rapidly developing field of digital healthcare [1–3]. Bioelectronic devices with high biocompatibility, flexibility, and degradability have been extensively exploited as wearable or implantable monitoring systems for human health, soft robotics, or human–machine interfaces. These devices are used to contact with soft, three-dimensional (3D) and moving tissues. Mechanically compliant substrates and passivation layers enable conformal contact and better adhesion with the tissue surfaces facilitating direct physiological recordings. In particular, synthetic or naturally derived polymers have been replacing conventional rigid and mechanically robust inorganic materials as the component of substrates [4]. They provide the electrodes with stretchability and conformability to follow the bending and elongation of biological tissues. Specifically, biologically derived biopolymers have attracted increasing attention due to their striking features such as high biocompatibility, biodegradability, sustainability, and natural abundance [5–7].

Above all, certain types of biopolymers have recently attracted significant research efforts for application in bioelectronics. For instance, polysaccharides such as alginate [8, 9] or chitin/chitosan [10, 11] as well as proteins such as silk fibroin [12, 13] have shown to provide not only a unique set of the aforementioned properties as biopolymers but also low immunogenic and inflammatory responses. Nevertheless, there is a limited number of solvents for dissolving or dispersing such biopolymers, which leads to technical difficulties in producing film substrates with uniform thicknesses and imparting the required electrical conductivity for the fabrication of bioelectronic devices. Although electrospinning or electrospraying methods have been conventionally employed to produce thin-film or micro/nanofibrous substrates, few types of solvents can be used for dissolving or dispersing those biopolymer-nanofibers [14]. Furthermore, there are challenges associated with the integration of precisely micropatterned electrodes with such materials based on standard lithography or accurate printing technologies. Often such approaches require expensive setups, harsh chemical or physical processes with etchants, and well-controlled ink preparation that are not compatible with biopolymers. Thus, in order to make biopolymers promising building blocks for biocompatible conductors, it is necessary to discover new fabrication processes and choose biopolymers that are not dissolved or disintegrated by those processes.

Here, we report a simple and versatile fabrication process to produce biopolymer-based electrodes without using lithography or printing techniques. The fabrication process only consists of the preparation of a hard mask and vacuum filtration. A rapid laser micromachining process is used to create masks with desired micropatterns using polyimide (PI) films. Since the vacuum filtration process is compatible with macromolecules, we choose chitosan nanofibers (CSNFs) for both, the substrate and the passivation layers. The CSNFs are derived from exoskeletons of crustaceans that exist in abundance and have attracted much interest in paper electronics and healthcare fields due to their excellent physical properties as well as their characteristics as biopolymers [15]. Owing to the high-aspect-ratio, nanofiber substrates with well-controlled thicknesses were formed simply by the vacuum filtration process. In addition, we show that this process is also suitable for fabricating micropatterns on CSNF paper with high-aspect-ratio conductive materials. As the electrodes, we use conductive macromolecules such as carbon nanotubes (CNTs) [16], metal nanowires (NWs) [17, 18], and poly (3,4-ethylenedioxythiophene):poly(styrenesulfonate) (PEDOT:PSS) [19] by taking advantage of their large size and compatibility with the filtration process [20–23]. As a proof-of-principle, we demonstrate the stimulation of the thoracic nerve of the locust using a CSNF/CNT paper-based electrode.

## Results and discussion

### Fabrication of stimulation electrodes

**Fig. 1:**
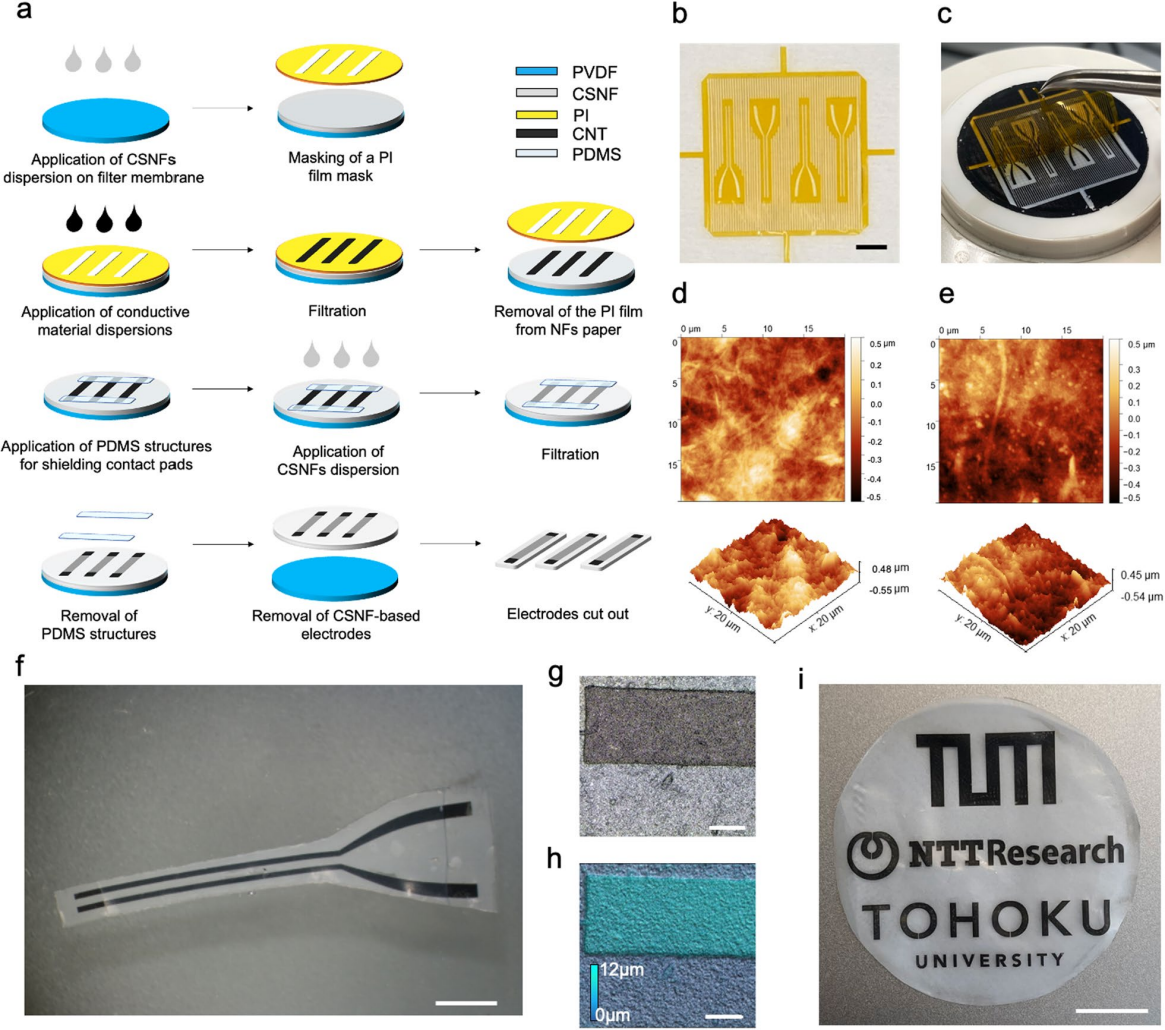
Fabrication process for CSNF-based electrodes for peripheral nerve stimulation. **a** Schematic of the fabrication process of CSNF paper electrodes. **b**, **c** Images of laser-patterned PI masks. PI masks are directly patterned by a laser ablation process (**b**), and removed gently after filtration to fabricate CNT micropatterns (**c**). **d**, **e** AFM images of the CSNF (**d**), and CNT network on CSNF (**e**). **f** An image of a CSNF paper electrode for in vivo experiments. **g**, **h** Laser-scanned 3D images of the surface topology of the electrode that is used for in vivo experiments. **i** An example of a patterned paper electrode without passivation. Scale bars: (b) 5 mm, (f) 2.5 mm (g, h) 100 µm (i) 1 cm

Figure 1a illustrates a simplified fabrication process of the CSNF paper-based electrodes for stimulation. At first, the CSNFs were produced by mechanically fibrillating chitosan powder with a water jet system. We dispersed CSNFs in water to 0.1 wt%. The filtration of the CSNFs dispersion through a polyvinylidene fluoride (PVDF) hydrophilic filter membrane produced the chitosan paper with high uniformity and optical transparency. The thickness of the paper was well controllable by adjusting the volume of the CSNF dispersion. Compared with the electrospinning process to produce CSNFs for cell scaffolding [24] or drug delivery [25], the mechanical fibrillation process via a water jet exhibits a lower risk of contamination during the extraction, thus yielding a higher material purity.

We used CNTs as the material for electrodes. CNTs have been used for stimulation electrodes due to their high charge transfer characteristics [26, 27], wide water window [16], and high biocompatibility [28]. To micropattern the electrodes, pores and grooves were fabricated in a polyimide (PI) film by using a laser ablation system (Fig. 1b). To fabricate flawless and conductive micropatterns on the nanofiber paper at a high yield, we mainly used 25-µm-thick PI masks. They are rigid enough to keep them flat during the handling process, enabling conformal contact between the paper and the mask. The filtration of a dispersion of CNTs through the PI film mask produced the filtrated electrode micropatterns on top of the CSNF paper. Subsequently, we removed the PI mask and placed polydimethylsiloxane (PDMS) masks to cover the areas for the contact pads. Afterward, the second filtration of the CSNF dispersion produced a passivation layer to cover the conductive patterns.

This all-filtration process using laser-patterned PI masks does not require standard lithography techniques for micropatterning the electrodes on top of the nanofiber paper. It should be noted that the timing of positioning and removing the PI film masks is critical for acquiring a good adhesion between CNTs and CSNFs. Since the dried CSNF paper caused weak attachment to the surface of the PI mask, we placed the mask directly after the first filtration of CSNF paper. Moreover, since the dried feedline and electrode structures caused better adhesion with CSNF paper, we carefully removed the masks several minutes after the filtration of the CNT dispersion (Fig. 1c). To facilitate the removal of the mask, we introduced additional patterns in the PI masks in addition to the electrode structures. These patterns reduced the adhesion between the paper and the PI for clean mask removal.

Atomic force microscopy (AFM) analysis of the CSNF substrates showed that individualized chitosan nanofibers of approximately 20–50 nm in diameter were entangled to form the paper surface (Fig. 1d). The root-mean-square (*R*_q_) surface roughness of the CSNF substrate was ~ 126 nm. The AFM image of a CNT/CSNF electrodes in Fig. 1e exhibited a surface roughness of *R*_q_ =  ~ 152 nm. Figure 1f shows a 2-probe CNT/CSNF electrode that was used for nerve stimulation. The average surface area of the patterned electrode material is 1.75 ± 0.02 mm^2^ (*n* = 24), and the 2 feedlines for the electrodes (200 µm in width and 300 µm in length) are sandwiched between 2 15 µm-thick CSNF layers. The 3D images of the surface topology of the electrode in Figs. 1g, h show the interface of the electrode with an intact CNT feedline on the nanofiber paper after removing the PDMS masks. Although PDMS was previously used for trapping patterned nanowires on filter membranes [17, 18], we used it here exclusively to cover the area for contact pads and electrodes during the following filtration of the nanofiber dispersion. Since the drying process caused deformation and curling of the unconstrained electrodes, the substrates were sandwiched between 2 glass slides and hot-pressed at about 832 Pa for several minutes to keep them planar. This fabrication process is compatible with arbitrary patterns (Fig. 1i).

### Electrical and geometric characterization of CNT/CSNF electrodes

**Fig. 2:**
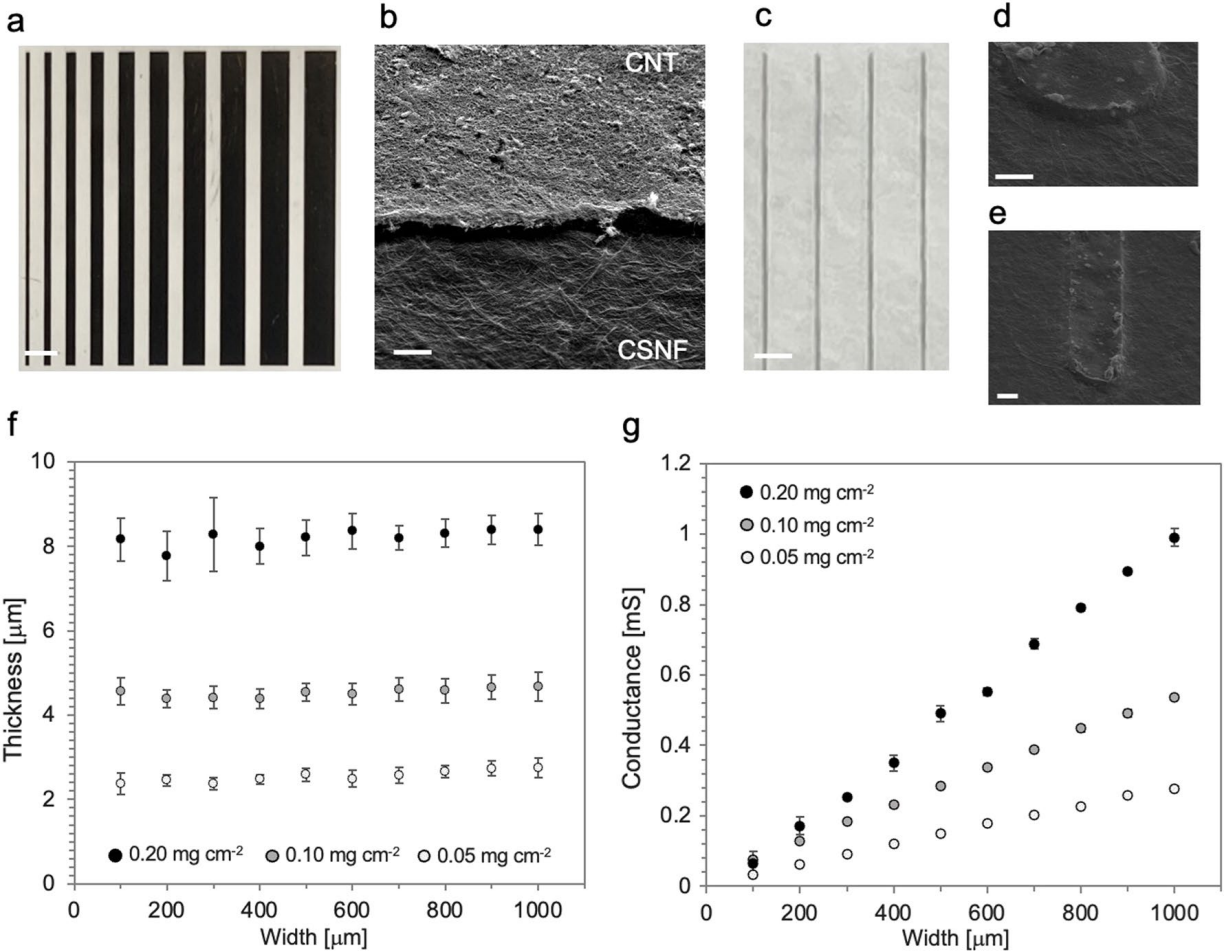
Characteristics of CNT feedlines embedded in CSNF papers. **a** CNT feedlines with 100-1000 µm in width and 1 cm in length, respectively. **b** An SEM image of the border between CNT networks and CSNF paper substrates. **c** CNT feedlines 25 µm in width. **d**, **e** SEM images of image (**c**). **f**, **g** The thicknesses and conductance of CNT feedlines dependent on the different surface densities (black dots: 0.2 mg cm^−2^, gray dots: 0.1 mg cm^−2^, white dots: 0.05 mg cm^−2^) and width of feedlines. The average thicknesses (*n* = 18, mean ± standard deviation) (f) and the conductance of CNT feedlines (*n* = 3, mean ± standard deviation) (**g**). Scale bars: **a**, **c** 1 mm, **b** 5 µm, **d**, **e** 10 µm

We performed electrical and geometric characterization of the CNT feedlines on the CSNF paper. To expose the electrodes during characterization, we skipped the process to form the passivation layers (Additional file 1: Fig. S1). Figure 2a shows CNT feedlines ranging from 100 µm to 1000 µm in width and 1 cm in length. The SEM image of the CNT feedlines (Fig. 2b) indicates that the CNT network was assembled on the nanofiber paper corresponding to the laser micropatterns in the PI mask. Additionally, we produced thin feedlines using CNTs. As shown in Figs. 2c–e, the minimum width of the feedline reached less than 25 µm, which is similar to the single spot size of laser ablation. The geometric resolution of the feedlines on top of the CSNF paper was determined by the accuracy of the laser system.

The average thickness and conductance versus the width of the CNT feedlines are plotted in Figs. 2f, 2g, respectively. The thickness of the feedlines is independent on the widths of the feedlines (Fig. 2f). This result suggests that CNTs were dispersed well enough to produce uniform CNT networks on CSNF papers regardless of the shape of the patterns. As expected, thicker CNT networks exhibited higher conductance. The changes scales almost linearly with the widths of the feedlines and the surface density of the CNT network (Fig. 2g). Thus, we confirmed that the conductance of the patterned CNT networks was simply controlled by the area and the amount of CNT dispersion. The conductivity of the CNT feedlines with 0.20 mg cm^−2^ was 1064 ± 170 S m^−1^ (*n* = 10, mean ± standard division), which is significantly higher than values reported in a previous study using PDMS substrates (173 ± 27 S m^−1^) [16]. The improvement in the conductivity probably originates from the direct patterning using the hard mask and the different substrate material. Generally, the transfer of high-aspect-ratio conductive material networks from a membrane filter to a substrate may cause mechanical damages to the electrodes—such as cracks—and deteriorate the conductivity of conductive material networks. Instead, the present fabrication process exploits micropatterning of conductive material networks directly on the substrate.

### Other conductive materials embedded in CSNF substrates

**Fig. 3:**
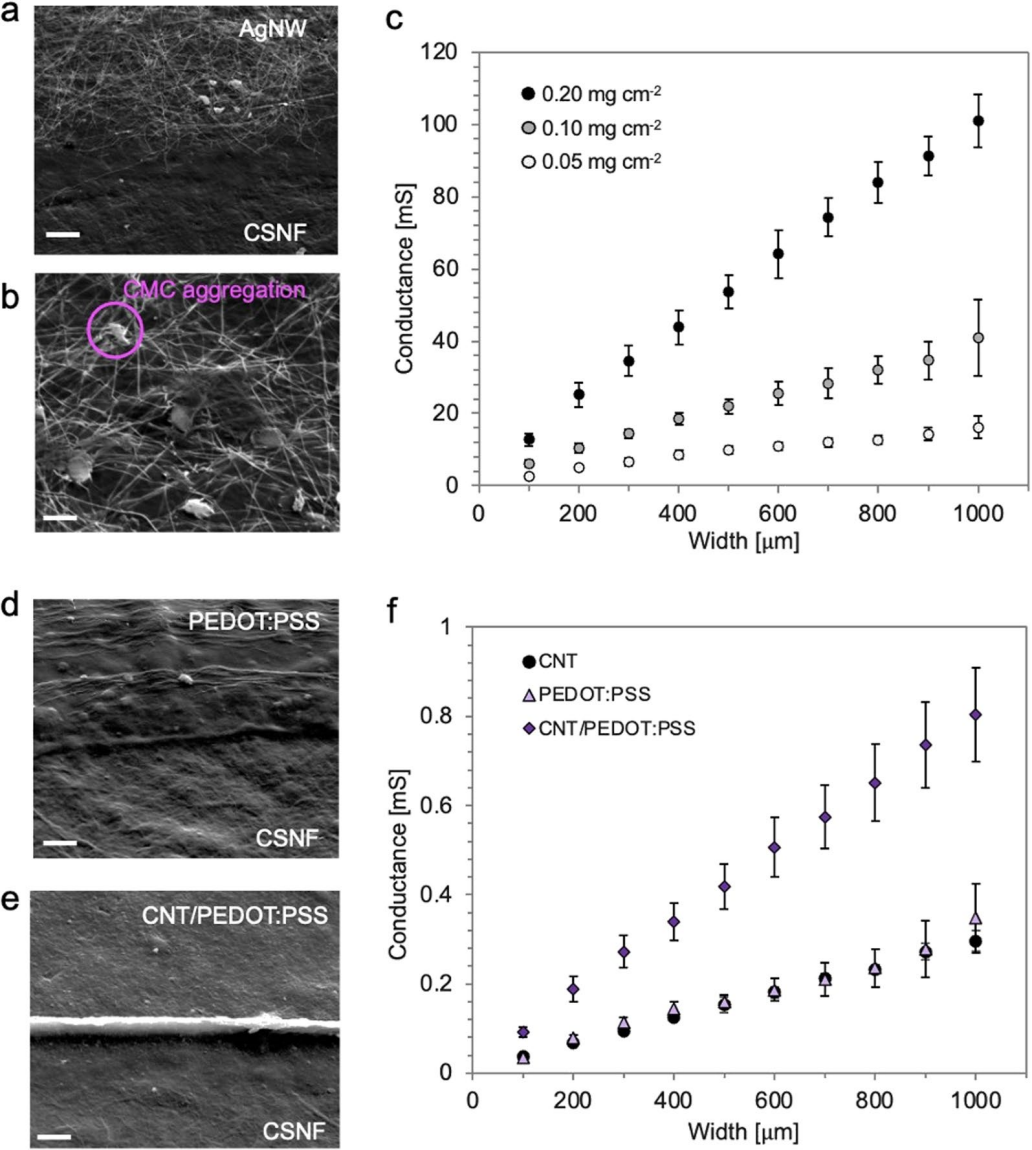
Characteristics of various conductive materials embedded in CSNF substrates. **a** SEM image of the border between AgNW networks and CSNF paper. **b** carboxymethylcellulose nanofiber aggregations entangled in AgNW networks. **c** Conductance of AgNW feedlines with different surface densities (black dots: 0.2 mg cm^−2^, gray dots: 0.1 mg cm^−2^, white dots: 0.05 mg cm^−2^, *n* = 6, mean ± standard deviation). **d**, **e** SEM images of a border between PEDOT:PSS and the CSNF substrate **d** and between CNT/PEDOT:PSS composites and the CSNF substrate. **f** Conductance of CNT (circle dots), PEDOT:PSS (triangle dots), and CNT/PEDOT:PSS composite feedlines (diamond dots). (*n* = 5, mean ± standard deviation). Scale bars: **a**, **d**, **e** 5 µm, (b) 2 µm

The presented filtration-based fabrication process is applicable for other types of conductive materials with high aspect ratio. The SEM images in Figs. 3a, b show silver nanowires (AgNWs) filtrated and assembled on the CSNF paper. Apparently, aggregations of carboxymethylcellulose (CMC) can be found among the AgNW networks (Fig. 3b). For the filtration of AgNWs, they were dispersed in water and CMC was added as a thickener and lubricant to keep the AgNW networks embedded and well attached with the CSNF substrate. Furthermore, the electrostatic interaction between CMC and CSNF enhances their mutual adhesion because CMC and CSNF are polyanions and polycations, respectively [29]. Owing to the CMC within the AgNW networks, we successfully produced a passivation layer on the AgNW micropatterns without detaching the patterned AgNWs. Figure 3c shows that the conductance of AgNW networks can be simply controlled by the area of the feedlines and the amount of AgNW dispersion similar to the CNT patterns. The conductance of AgNW patterns increased roughly by two orders of magnitude compared with that of CNT networks. Although AgNWs have low biocompatibility due to release of silver ions [30], this facile filtration-based patterning process is applicable for large-scale paper electronics [31] or stretchable electronics [32].

We further investigated the applicability of this process for patterning conductive polymer PEDOT:PSS on CSNF. PEDOT:PSS is one of the most promising conductive polymers for bioelectronics [33, 34] and flexible devices [35, 36]. The filtration of pristine aqueous PEDOT:PSS solution through the PI masks led to the formation of micropatterns on CSNF substrates. The SEM image in Fig. 3d shows the relatively smooth surface of PEDOT:PSS that was deposited. We observed some wrinkles on the surface of feedlines in parallel to their longer axis, close to the edges of feedlines (Additional file 2: Fig. S2b). In contrast, PEDOT:PSS was not trapped on the PVDF membrane without CSNFs, implying that pores of the CSNF paper act as a filter to capture pristine PEDOT:PSS. The average size of PEDOT:PSS particles were found to range from 50 to 600 nm using dynamic light scattering measurements, which enables the particles to be trapped in the paper substrate [37]. Conventionally, PEDOT:PSS was directly filtrated to produce the conductive film using a filter membrane by making the agglomeration of PEDOT:PSS nanoparticles large in size with specific solvents [38, 39]. On the other hand, paper substrates have been used for filtrating materials such as gold nanoplatelets [22], rhodamine [22], and SIV viruses [40]. Thus, it is reasonable that this filtration process is compatible with a range of materials. In this study, the micropatterns of PEDOT:PSS were generated in a chemical-free and facile manner without further modification using only pristine PEDOT:PSS and deionized water.

The simultaneous filtration of the mixture of CNT and PEDOT:PSS alters both surface morphology and conductivity, compared with that of pristine CNT or PEDOT:PSS. An SEM image in Fig. 3e shows the surface of feedlines of CNT/PEDOT:PSS composites. The structures were deposited and assembled on top of the nanofibers without phase separation during the filtration. The surface of CNT/PEDOT:PSS was relatively smooth compared to bare CNTs shown in Fig. 2b. In addition, there were less cracks and wrinkles on the surface compared to pristine PEDOT:PSS (Additional file 2: Fig. S2c). Furthermore, addition of CNTs to PEDOT:PSS improves the conductivity. As shown in Fig. 3f, the conductance of PEDOT:PSS feedlines with a density of 0.4 mg cm^−2^ was almost the same as the one of CNT networks with 0.05 mg cm^−2^. When we filtrated the mixture of CNT and PEDOT:PSS with a density of 0.4 mg cm^−2^ and 0.05 mg cm^−2^ respectively, the conductance was three times higher than that of pristine CNT networks or PEDOT:PSS. Both the non-linear increase of conductivity and the smooth surface are in good accordance with previous reports that suggest that the conductance of CNT composites is not proportional to the concentration of CNTs [41, 42]. As previously reported [41], the CNT/PEDOT:PSS composite exhibited a smooth surface and improved mechanical characteristics that include lower tensile strength and larger elongation at breaks thanks to the interaction of polymers with CNTs.

### Mechanical and electrochemical characterization of CNT/CSNF electrodes

**Fig. 4:**
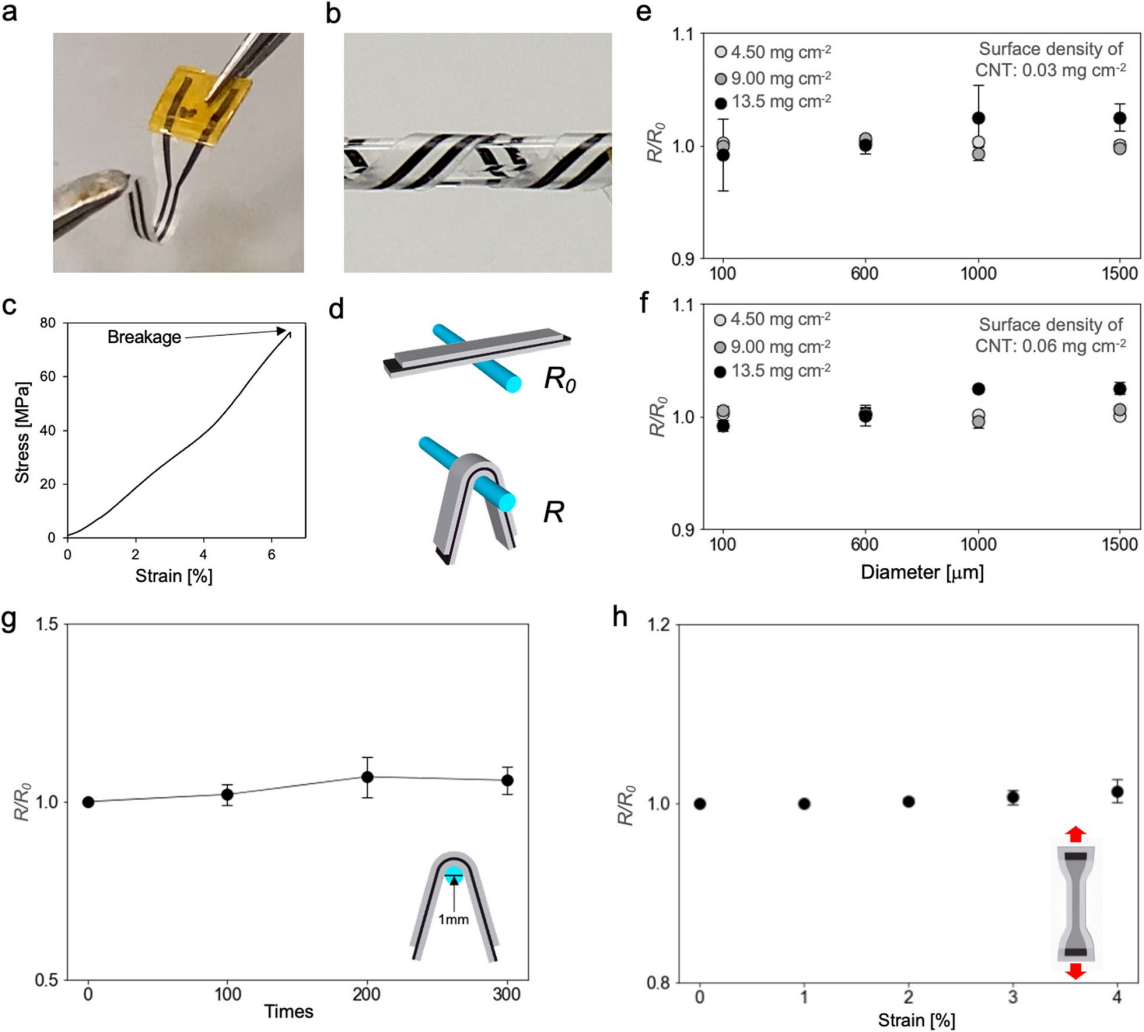
Changes in resistances against mechanical deformation with CNT/CSNF electrodes. **a** Image of a CNT/CSNF electrode fabricated for in vivo experiments. **b** The electrode was bent and wrapped around a glass rod (1 mm in diameter). **c** An exemplary stress–strain curve of a CNT/CSNF electrode. **d** Schematics of resistance measurements with CNT/CSNF electrodes before and while being wrapped around the glass rods. **e**, **f** Relative resistance of CNT/CSNF electrodes with different surface density of CNTs and CSNF wrapped around glass rods (*n* = 3, mean ± standard deviation). The surface density of CNT are 0.03 mg cm^−2^ (**e**) and 0.06 mg cm^−2^ (**f**), respectively, and the electrodes have three varieties of CSNF density with 4.5 mg cm^−2^ (white dots), 9.0 mg cm^−2^ (gray dots) and 13.5 mg cm.^−2^ (black dots). **g** Relative resistance of CNT/CSNF electrodes in dependence on bending cycles (n = 3, mean ± standard deviation). **h** Relative resistance of CNT/CSNF electrodes during strain applicatioin (n = 4, mean ± standard deviation)

We performed additional mechanical and electrochemical characterization of the CNT/CSNF electrodes for potential applications as bioelectronic interfaces. First, we investigated the mechanical characteristics of the fabricated CNT/CSNF electrodes (Fig. 4a). The CNT/CSNF electrodes are highly flexible so that they can be bent and wrapped around a glass rod by twisting them (Fig. 4b). This flexibility is one of the important properties of bioelectronic devices to enable mechanical deformation of itself and endow conformal contacts of bioelectronic interfaces with biological tissues [43]. The maximum strain until breakage of the electrodes was 6.1 ± 1.8 [%] (n = 4) (Fig. 4c). This result shows that the electrode can endure a relatively large strain compared to other cellulose nanofiber substrates [44]. To evaluate the mechanical properties of the electrode for potential application as bioelectronic interfaces, we conducted additional experiments to investigate the relative resistance of the electrode under mechanical deformations. First, we measured the resistance of the electrodes before and while being wrapped around the glass rods (Figs. 4d). Furthermore, we prepared the CNT/CSNF electrodes with six-different surface densities to see if the surface densities of the electrodes affect the changes in resistance caused by the application of this mechanical deformation. The observed change in resistance (*R*/*R*_o_) was not strongly affected by different surface densities (Fig. 4e, f). Moreover, the values of *R*/*R*_o_ did not change severely on the diameter of the glass rod regardless of different surface densities. This result corresponds to a previous report in which the resistance of the AgNW networks embedded in a cellulose nanofiber paper did not strongly change during folding multiple times [30]. Thus, we assume that the entangled CNT network structure leads to sufficient flexibility to maintain its electrical conductivity during mechanical deformation.

Second, we measured the resistance of CNT/CSNF electrodes under repetitive bending (Fig. 4g). The observed relative change in resistance of the electrodes during multiple bending cycles was rather low compared to e.g. AgNW-embedded TEMPO cellulose nanofiber paper [45]. Also, the resistance was rather stable under moderate strain (Fig. 4h) in comparison to e.g. Ti/Au evaporated cellulose nanofibers [44]. We attribute the stability of relative resistance to the passivation layer for the electrodes, which embeds the CNT network into CSNF substrate and passivation layer.

**Fig. 5:**
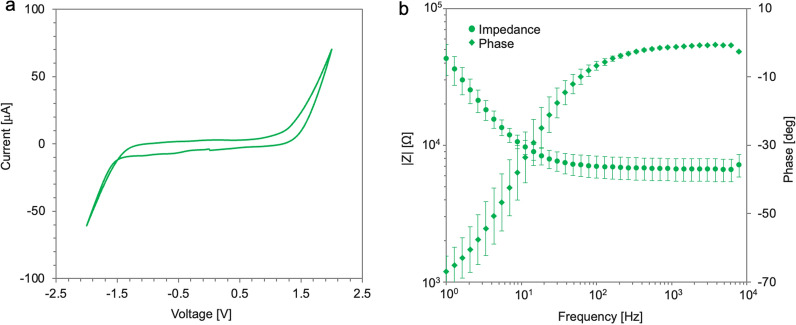
Electrochemical characterization of CNT/CSNF electrodes. **a** Cyclic voltammograms for the CNT/CSNF electrodes sweeping from -2 V to 2 V vs Ag/AgCl to determine the water window. **b** Impedance spectroscopy for the electrodes (*n* = 8, mean ± standard deviation)

Next, we investigated the electrochemical characteristics of CNT**/**CSNF electrodes. Before the experiments, we covered the lead of the electrodes with polyurethane (PU) for passivation. PU has been widely used for insulating implantable electrodes while preserving biocompatibility and flexibility [46]. The results of the cyclic voltammetry (CV) measurement in the range between − 2.0 V and 2.0 V vs Ag/AgCl are shown in Fig. 5a. The water oxidation and reduction limits of the CNT electrodes were found to be about + 1.5 V and − 1.5 V, respectively. The water window of our CNT electrodes corresponded to the water window of previous work using CNT as a conductive material on electrodes [16, 27]. This large water window has the advantage that the electrode can be polarized to high potential without irreversible faradaic reactions that cause undesirable side effects, such as pH changes, electrode degradation, and tissue damage [47–50]. As shown in Fig. 5b, the impedance values of the electrodes at 1.0 kHz were 6.7 ± 1.3 kΩ (*n* = 8, mean ± standard deviation). The impedance and phase of CNT/CSNF electrodes in PBS are similar to the results reported in previous research confirming their suitability for neuronal stimulation electrodes [16, 51].

### In vivo experiments

**Fig. 6:**
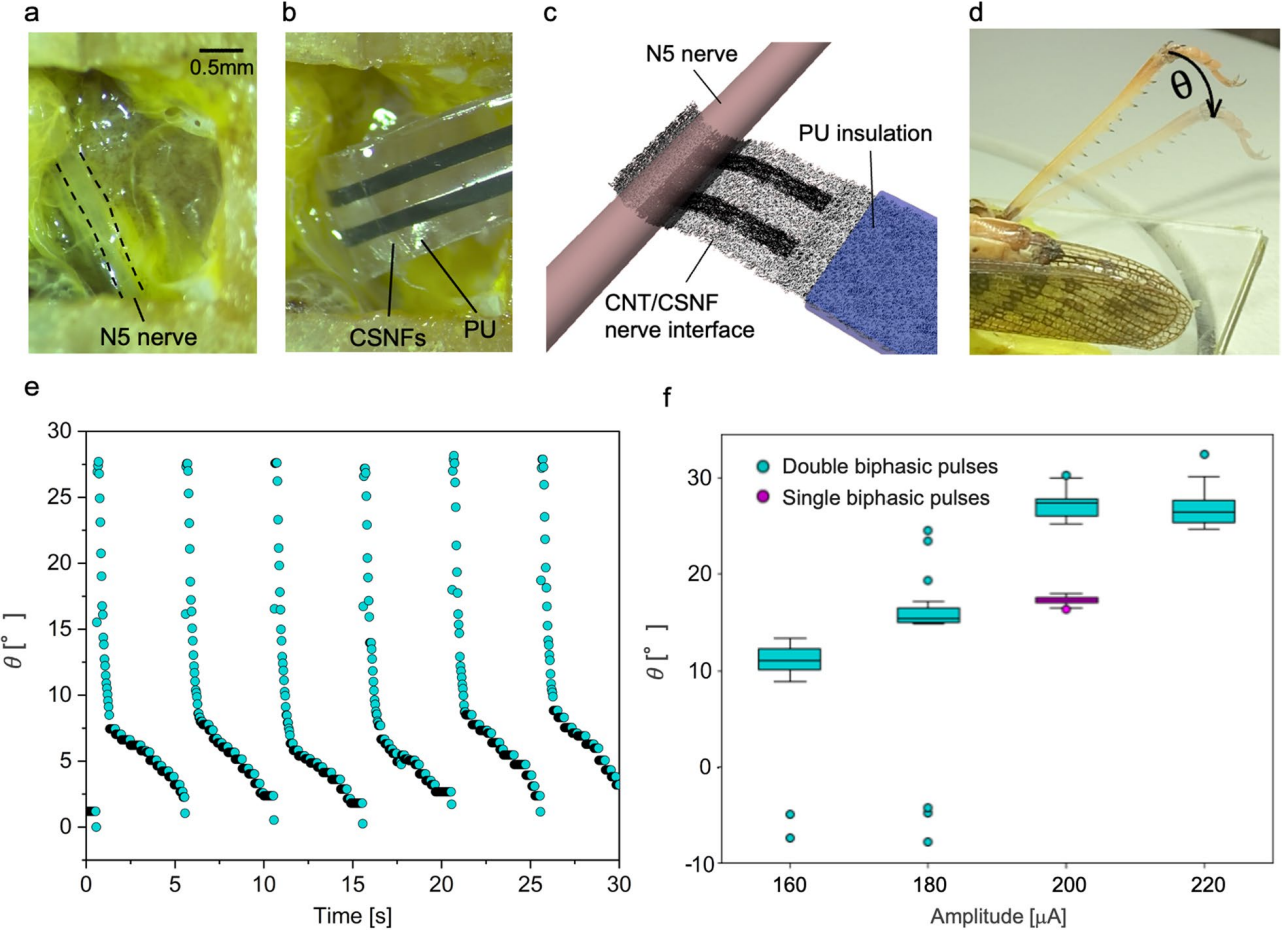
in vivo implantation of the electrodes using locusts. **a**, **b** Image of the N5 of the locust. **b** A CNT/CSNF electrode interfaces with the N5 of the locust. **c** A schematic drawing of the interface between a CNT/CSNF electrode and an N5 of a locust. **d** An image showing the locust-tibia extension with the corresponding change in angle. **e** The trace of the angle measured during application of 220 µA double biphasic pulses every 5 s. **f** The angle of the locust-tibia extensions caused by stimulations (*n* = 15, mean ± standard deviation)

As a proof of principle, we conducted in vivo experiments with a locust, *Locusta migratoria*. The locusts have been used as a model organism in neurobiological research due to their intriguing and easily accessible nervous system [52, 53]. The target tissue of the locust in our experiments is the rapid extensor nerve (N5) in the metathoracic ganglion since the N5 innervates the extension and flexion of the hind legs [54] (Fig. 6a). As shown in Fig. 6b, we inserted and attached the electrode to the N5 of the locust. It can be seen that the PU insulator was not in contact with the N5 of the locust. The schematic in Fig. 6c represents the interface between the N5 and the electrode. Throughout the surgery of locusts, insertion of the electrodes and stimulation experiments, we did not observe any critical damages to the nerve and its surrounding tissues.

In this configuration, we stimulated N5 of the locust by applying biphasic current pulses. Each time when biphasic current pulses were applied to the N5 of the locust, we observed the extension of the locust tibia (Fig. 6d). Figure 6e shows the trace of the locust tibia while biphasic pulses were applied every 5 s. We observed stable extensions followed by slow flexions by the contraction of flexor muscle with each stimulation. Each extension was observed within a few tenth of milliseconds, which is closely identical to the results of locust’s tibial extensions regulated by fast tibiae motor neuron [55]. This result confirms the possibility to apply stimulation by CNT/CSNF electrodes eliciting neural activities to extend the locust tibia.

The angles of the extension changed depending on the amplitude of the applied current pulse and the number of pulses (Fig. 6f). We applied double biphasic current pulses composed of two consecutive single biphasic pulses. The angle of extension increased with stimulation amplitudes up to 200 µA. This result implies that 200 µA of double biphasic pulses was high enough to elicit the maximum leg extension. During application of 160 and 180 µA of double biphasic pulses, we sometimes observed fast flexions or contractions of the tibia, as opposed to the usual extensions, leading to negative angles. These phenomena might occur because of random biological actions of the locust. Also, we attributed them to the displacement of the electrode from the nerve caused by some motion of the locust during the experiment. Furthermore, not all the current applied reaches the target tissue depending on the precise electrode location with respect to the nerve. To acquire better contact and yield stable stimulations during biological movement, we believe this electrode should be developed into a cuff-electrode to provide a more secured interface between electrode and biological tissues [46]. Next, we applied single biphasic pulses at 200 µA and found that the angle of extension was lower than the same amplitude of double biphasic pulses. This result shows that intervals of each biphasic pulse contribute to modulating the angle of leg extensions, which corresponds to previous results about modulating leg movements of locusts by changing intervals of each pulse [56]. Overall, we successfully stimulated the N5 of the locust with the CSNF/CNT electrode, demonstrated high biocompatibility of the implanted electrodes, and modulated the angle of the extension by changing the amplitude and the number of biphasic pulses applied in each stimulation.

## Conclusion

We established a fabrication process to micropattern conductive materials on CSNF substrates by vacuum filtration using laser-patterned PI masks. We demonstrated that this process is applicable to high aspect-ratio conductive materials such as carbon nanotubes and silver nanowires as well as conductive polymers based on PEDOT:PSS. Our results provide guidelines for fabricating micropatterns of conductive materials on nanofiber papers. Finally, using this versatile fabrication method, we fabricated flexible CNT/CSNF electrodes for peripheral nerve stimulation and performed in vivo experiments with the N5 of the locust. We modulated the extension of the locust leg by applying biphasic current pulses. We believe that the presented approach will be useful for the development of future flexible bioelectronic devices based on CSNF paper substrates.

## Materials and methods

*Preparation of CSNF papers*: CSNFs were used for substrates and passivation layers of paper electrodes. The material was generated from chitosan powder derived from crab shell chitin. The dry crab shell was decalcified, deproteinized, and deacetylated to convert it to chitosan powder. The obtained chitosan powder displayed an average degree of polymerization of 480 and a degree of acetylation of 93.8% estimated by 1H-NMR. The chitosan powder was suspended in water using a homogenizer (Ultra Turrax, IKA) for 5 min and a 2 wt% slurry was prepared. Afterwards, the slurry was mechanically fibrillated using a water jet system (Star Burst, HJP-25005, Sugino Machine Limited) equipped with a ball-collision chamber, at 2000 bar with a 0.14 mm nozzle to prepare CSNFs. Each nanofiber is 20–50 nm in diameter and several micrometers in length.

2 wt% solutions of CSNFs were mixed with deionized water to dilute to 0.1 wt% and dispersed by using an ultrasonic homogenizer (generator: GM 2200.2; transducer: UW 2200; booster: SH213; Sonotrode: KE76, Bandelin) for 40 min. To produce CSNF paper, 0.1 wt% solutions of CSNF dispersion were filtrated through a filter membrane (Durapore, 0.22 µm pore size, polyvinylidene fluoride (PVDF) membrane, φ = 47 mm) by a vacuum pump (MD 1, 1.5 mbar, Vacuubrand GmbH). To produce 15 µm thick CSNF paper, 15 mL of 0.1 wt% CSNF solution was filtrated.

*Micropatterning of electrodes*: On top of the CSNF paper, two types of conductive materials were micropatterned by the filtration method. To prepare the CNT dispersion, 1.43 mg of CNT powders (Hanos MWCNT M-95, entangled, 93–97% purity, Hanwha Chemical) and 200 mg sodium dodecylbenzene sulfonate (Sigma Aldrich) as the surfactant were mixed with 100 mL deionized water and homogenized for 50 min using the ultrasonic homogenizer. The dispersion solution of AgNWs was prepared by adding AgNWs (60 nm in diameter, 10 µm long, Sigma-Aldrich) to 100 mL of diluted water. In advance, 350 mg carboxymethyl cellulose nanofiber (Sugino Machine) was dissolved in the distributed water and homogenized for 35 min using the ultrasonic homogenizer. Before the filtration, the suspension of AgNWs in isopropyl alcohol was mixed with the homogenized dispersion of carboxymethyl cellulose nanofibers to a concentration of 3.93 mg mL^−1^ and vortexed briefly.

To micropattern the electrodes, we used 25 µm-thick polyimide films as the hard masks. Desired patterns of pores and grooves were fabricated in polyimide films by using a laser cutter (3-Axis UV Laser marker, MD-U1000C, Keyence). The design of micropatterns was created with 2D CAD software (AutoCAD2022, Autodesk). The patterned polyimide films were put on the CSNF paper on the PVDF membranes. Then, the dispersion of CNT and AgNWs was filtered through the polyimide film mask to yield a surface density of 0.20 mg cm^−2^. After the filtration, the polyimide mask was peeled off carefully.

To form the passivation layer, CSNF dispersion was filtered to cover the conductive material patterns. Before the filtration, the PDMS slabs (Sylgard 184, Dow Chemical) were placed in order to expose the areas of contact pads and tips for the interfaces with nerve tissues. 15 mL CSNF dispersion was filtered to produce a 15-µm-thick passivation layer of CSNF. After the filtration, the PDMS slabs were removed mechanically with tweezers, and the multilayered electrodes were cut out by scissors. After drying, electrodes were sandwiched between glass slides with 18.75 cm^2^ areas and pressed by metal weights (160 g) for 5 min on a hotplate (RH basic 2, IKA). This enabled the hot press to flatten the electrodes with a pressure of 836 Pa. Afterwards, we pasted some Kapton tapes on the backside of electrodes to get a better connection with the connecter of the circuit for in vivo experiments.

*Characterization of micropatterned electrodes*: The polyimide mask has feedline patterns with 100–1000 μm in width and 1 cm in length, creating the corresponding electrodes on the filtered CSNF paper. The surface morphology was measured using a Bruker Dimension Icon AFM with a Nanoscope V Controller. The roughness was calculated using the software (Gwyddion AFM). Thicknesses of different surface densities of the feedlines were measured by a confocal laser microscope (3D Laser Scanning Microscope, VK-X200, Keyence). Each CNT feedline has the same density to calculate its thickness. For the mechanical characterization, we used a tensile tester (Universal Testing Machine, TesT GmbH). The samples were stretched with constant velocity of 10 mm min^−1^ until breakage and the force was recorded against the displacement. The stress and strain were measured and calculated by using the software (TesTWinner 950). The electrodes were observed using optical microscopy and scanning electron microscopy (SEM). Optical images were acquired with the microscopes (Carl Zeiss) and a digital camera (EOS8000D, Canon) with a microscope adapter (NY1S, Mecan). SEM images were acquired by collecting secondary electrons on an SEM (JOEL) working at 15 keV. Before SEM imaging, the surfaces of paper electrodes were gold metalized in a metal sputter coater (Med020, BalTec).

We measured the resistance of each feedline on nanofiber papers by using a digital multimeter (TY520, Yokogawa) and calculated each feedline’s conductance. Subsequently, we prepared electrodes of six different thicknesses by controlling the amount of dispersion for filtration. The amounts of nanofiber dispersion for the substrate of electrodes were about 10, 20, and 30 mL to about 20 and 40 mL of CNT dispersion. Samples were approximately 20 mm long and 5 mm wide. For this evaluation, we prepared glass rods of four different diameters (100, 600, 1000, and 1500 μm). We measured the resistances of each electrode before and while making them bend along the glass rods. Afterward, we calculated *R*/*R*_o_ for each electrode; *R*_o_ and *R,* respectively, refer to resistance before and while bending.

For the electrochemical characterization, cyclic voltammetry (CV) and electrochemical impedance spectroscopy (EIS) measurements were performed by a potentiostat (PalmSens4, PalmSens BV). The CNT/CSNF electrodes with single feedlines were immersed in phosphate buffered saline (pH = 7, *ρ* = 0.63 µ m, Sigma-Aldrich) at room temperature. Then, we measured them in a three-electrode setup with an Ag/AgCl reference electrode (RE-6, Basi, West Lafayette) and platinum meshes with large surface areas as a counter electrode. The open circuit potential between the CNT/CSNF working electrode and a Ag/AgCl reference electrode was measured to be approximately − 0.21 V. Cyclic voltammetry was applied in three different ranges for different purposes. To calculate charge storage capacity, CV was performed between − 0.5 V and 0.5 V vs Ag/AgCl for three cycles. For characterization of the electrochemical water window, CV was performed between − 2.0 V and 2.0 V vs Ag/AgCl for three cycles to determine the water oxidation and reduction limits for the CNT/CSNF electrodes. In all CVs, the start and finish potential were set to 0 V vs Ag/AgCl, and the measurement started with a negative sweeping direction. EIS was performed in the frequency range of from 1 to 10^4^ Hz at 10 mV (rms) on a single feed line of CNT electrodes.

﻿﻿In vivo﻿﻿ surgery: The locusts that we used were kept in a plastic terrarium and fed with grasses. Before the surgery, we anesthetized locusts by keep them at 2℃ for around 30 min for immobilization. Afterwards, we fixed the locusts on plates using modeling clay with the ventral side upward at room temperature and humidity. Then, we would remove the cuticle of the ventral thorax and carefully cut the muscles attached to it. Subsequently, we removed the air sacs and the trachea so that we could easily treat the N5 that innervates the hind legs. Finally, we would carefully insert the interface part of the electrode inside the locust and contact the electrode with the N5. Surgeries were performed using a microscope.

In vivo experiment: The contact pads of the electrode were connected to the self-made electrical setup. Then, we applied biphasic current pulses that have a single amplitude between two feedlines on the electrode every 5 s up to 15 times by using a modular electrophysiology data acquisition system (RHS, Intan technologies) and its software (RHX, Intan technologies). Each cathodic and anodic pulse has a duration of 500 µs, and the amplitude of the current pulse ranged from 140 to 220 µA with each 20 µA step. The interval of each entire biphasic current pulse was 10 ms. While applying the current pulses, we recorded the extension of the locust leg by filming with a camera from the side view of the locust.

## Supplementary Information

**Additional file 1: Figure S1.** Fabrication process of electrodes for evaluation of electrical and geometric characterization. The electrodes are composed of CSNFs and patterned conductive materials including CNTs, AgNWs and PEDOT:PSS without the passivation layers.

**Additional file 2: Figure S2.** SEM images of surface topology of conductive materials on CSNF substrate. (a) The boundary between CNT networks and CSNF substrate where CNT networks partially detached. (b) Wrinkles on the surface of PEDOT:PSS micropatterns on the CSNF substrate. (c) The surface of CNT/PEDOT:PSS composite micropattern on the CSNF substrate. Scale bars: (a) 5µm, (b) 2µm, (c) 2µm.

## Data Availability

All data needed to evaluate the conclusions in the paper are present in the paper and/or the Supplementary Materials. Additional data related to this paper may be requested from the authors.
