# Supplementary figures and images for: Filtration-processed biomass nanofiber electrodes for flexible bioelectronics
Source: J Nanobiotechnology. 2022 Nov 19;20:491. doi: 10.1186/s12951-022-01684-3 (PMC9675094; doi:10.1186/s12951-022-01684-3)

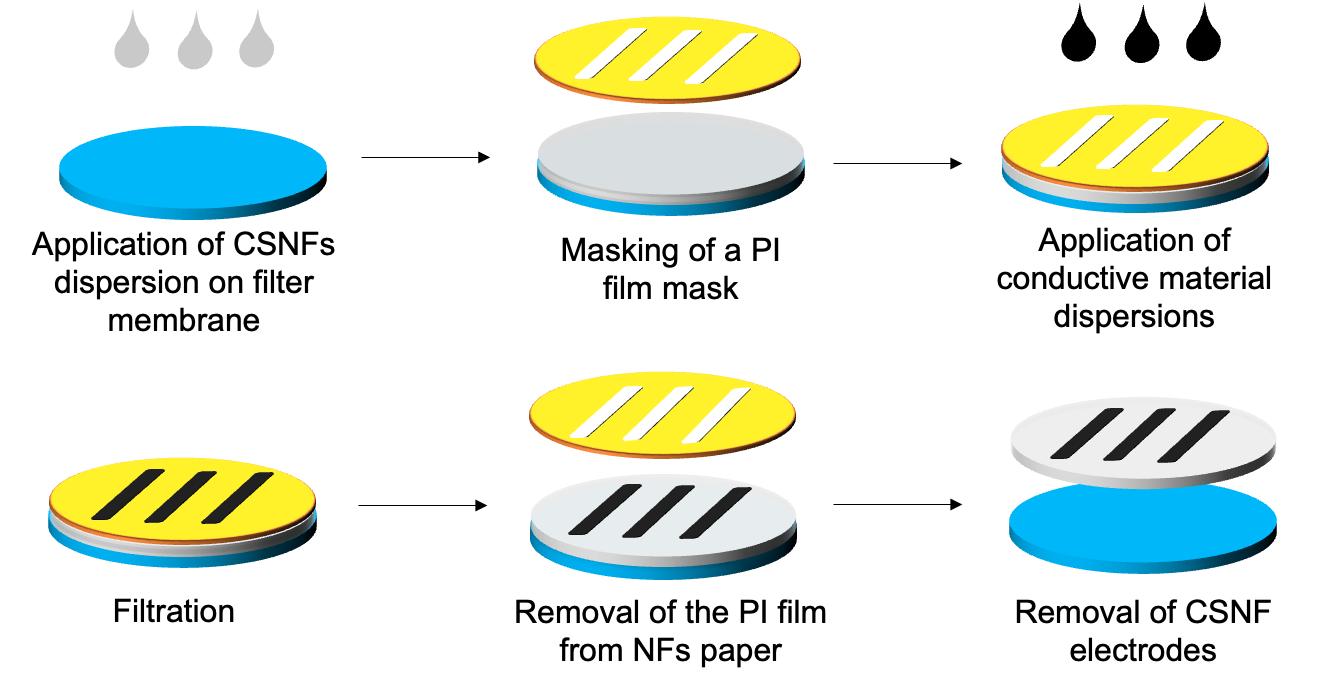

Supplement: Supplementary file 1 — Additional file 1: Figure S1. Fabrication process of electrodes for evaluation of electrical and geometric characterization. The electrodes are composed of CSNFs and patterned conductive materials including CNTs, AgNWs and PEDOT:PSS without the passivation layers. [file 12951_2022_1684_MOESM1_ESM.png]

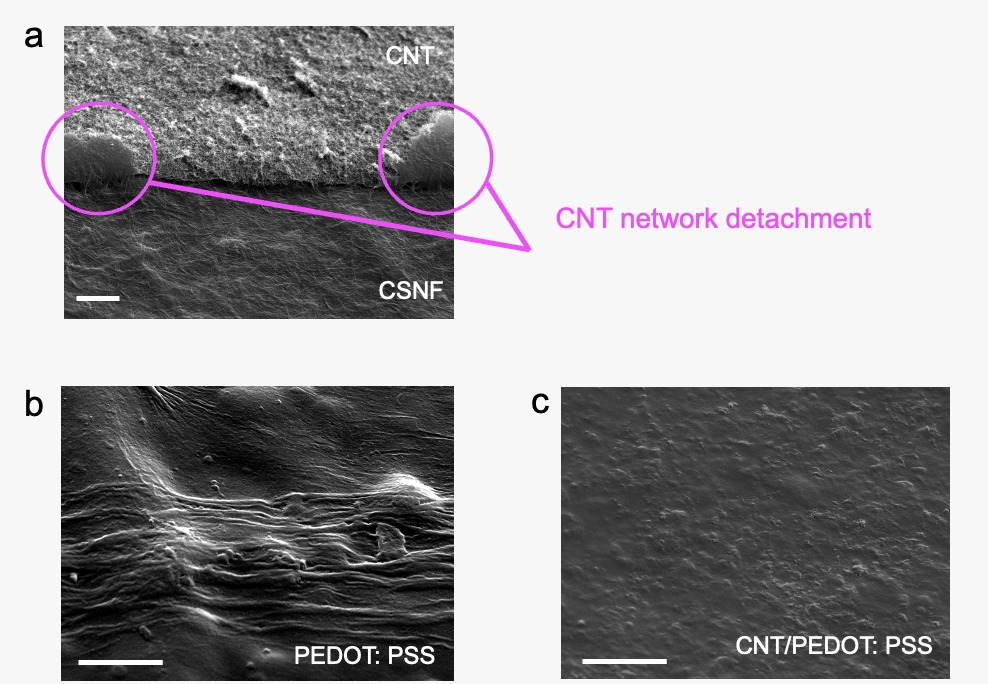

Supplement: Supplementary file 2 — Additional file 2: Figure S2. SEM images of surface topology of conductive materials on CSNF substrate. (a) The boundary between CNT networks and CSNF substrate where CNT networks partially detached. (b) Wrinkles on the surface of PEDOT:PSS micropatterns on the CSNF substrate. (c) The surface of CNT/PEDOT:PSS composite micropattern on the CSNF substrate. Scale bars: (a) 5µm, (b) 2µm, (c) 2µm. [file 12951_2022_1684_MOESM2_ESM.jpg]
